# Unraveling Oxygen Vacancies Effect on Chemical Composition, Electronic Structure and Optical Properties of Eu Doped SnO_2_

**DOI:** 10.3390/nano14201675

**Published:** 2024-10-18

**Authors:** Maxim A. Mashkovtsev, Anastasiya S. Kosykh, Alexey V. Ishchenko, Andrey V. Chukin, Andrey I. Kukharenko, Pavel A. Troshin, Ivan S. Zhidkov

**Affiliations:** 1 Institute of Physics and Technology, Ural Federal University, Mira Str. 21, 620002 Yekaterinburg, Russia; 2 M.N. Mikheev Institute of Metal Physics of Ural Branch of Russian Academy of Sciences, S. Kovalevskoi Str. 18, 620108 Yekaterinburg, Russia; 3 Zhengzhou Research Institute of HIT, Longyuan East 7th, 26, Jinshui District, Zhengzhou 450003, China; 4 Federal Research Center for Problems of Chemical Physics and Medicinal Chemistry RAS, Semenov ave, 1, 142432 Chernogolovka, Russia

**Keywords:** XPS, electronic structure, europium, luminescence

## Abstract

The influence of Eu doping (0.5, 1 and 2 mol.%) and annealing in an oxygen-deficient atmosphere on the structure and optical properties of SnO_2_ nanoparticles were investigated in relation to electronic structure. The X-ray diffraction (XRD) patterns revealed single-phase tetragonal rutile structure for both synthesized and annealed Eu-doped SnO_2_ samples, except for the annealed sample with 2 mol.% Eu. The results of X-ray photoelectron spectroscopy (XPS) emphasized that europium incorporated into the SnO_2_ host lattice with an oxidation state of 3+, which was accompanied by the formation of oxygen vacancies under cation substitution of tetravalent Sn. Moreover, XPS spectra showed the O/Sn ratio, which has been reduced under annealing for creating additional oxygen vacancies. The pulse cathodoluminescence (PCL) demonstrated the concentration dependence of Eu site symmetry. Combination of XRD, XPS and PCL revealed that Eu doping and following annealing induce strongly disordering of the SnO_2_ crystal lattice. Our findings provide new insight into the interaction of rare-earth metals (Eu) with host SnO_2_ matrix and new evidence for the importance of oxygen vacancies for optical and electronic structure formation.

## 1. Introduction

Wide bandgap dielectrics, such as SnO_2_, TiO_2_, ZnO, etc., have found wide application in various optical and magnetic semiconductors [1,2], antibacterial coating [3] and sensing devices [4]. In recent years, significant attention has been paid to SnO_2_ due to its high optical transparency (band gap of 3.5–3.8 eV), *n*-type electrical conductivity, and adjustable optical and magnetic properties [5,6,7]. One of the most important applications of SnO_2_ is the charge transport layer in perovskite-based solar cells (PSC) [8], as well as solar-blind ultraviolet photodetector devices [9,10]. However, application in optical systems imposes certain requirements on the atomic and electronic structure of tin dioxide. Thus, possible defects (e.g., oxygen vacancies) can hinder the charge transfer during operation of PSC. In addition, it has been shown that lead-based hybrid perovskites can chemically react with tin dioxide, with partial oxidation of the former due to the migration of oxygen ions [11]. On the other hand, since SnO_2_ is a typical *n*-type semiconductor with some oxygen vacancies, a Schottky counter barrier is formed, which is facilitated by Fermi level pinning and surface states or defects [10]. Theoretical and experimental studies of SnO_2_ also showed that point defects in the host material contribute to vacancy-induced effects, which can affect device performance and stability [12,13,14].

To date, many studies have been devoted either to the influence of vacancies or doping with 3*d* metals on the optical and magnetic properties and electronic structure of SnO_2_. At the same time, doping with rare-earth (RE) elements (4*f* metals) makes it possible to combine the specified optical and magnetic properties in one material [15,16]. In particular, doping allows one to adjust the position of the band edges for better charge transport in multilayer solar cells. In addition, the literature does not pay much attention to the possible relationship between optical properties, electronic structure, and vacancies in these materials [17,18]. One of the most popular RE elements for doping semiconductor matrices is Eu [19,20]. Thus, in relation to solar panels, Eu was previously used to dope hybrid perovskites [21], which led to greater tolerance towards defect creation as well as to partial passivation of the surface, which is extremely important for interface reactions in the SnO_2_/hybrid perovskite system. However, if we are talking about doping the SnO_2_ matrix with Eu ions, then several unknown points arise that require further research. Thus, 4*f* metal ions in wide band gap semiconductor matrices, as a rule, replace Sn^4+^ ions of the host matrix. At the same time, doping of Eu in the 3+ charge state will cause a change in the local atomic and electronic structure, which in turn should be reflected in a change in the optical properties. In addition, the possible formation of vacancies in the structure of the host material will also have a significant effect on the optical properties and electronic structure. It is important to note that the concentration of doping elements will also have a significant effect on the material properties [22,23], which can be expressed in the formation of secondary phases (such as Eu_2_O_3_ or Eu_2_Sn_2_O_7_) [18].

Thus, the aim of this work was to reveal the influence of the oxygen vacancies and the Eu doping on the optical properties and electronic structure of SnO_2_.

## 2. Experimental Details

Aqueous solutions with an adjusted tin and europium ratio were prepared by dissolving SnCl_4_·5H_2_O and EuCl_3_·6H_2_O salts in water. Ammonia aqueous solution NH_4_OH with a concentration of 3 mol/dm^3^ was used as a precipitant. Co-precipitation was performed by simultaneously dosing an ammonia solution and a salt solution into a reactor, in which a constant pH = 3 was provided by controlling the feed rate of the ammonia solution. Before starting the co-precipitation, a solution of water and ethylene glycol with a ratio of 1:1 by volume was placed in the reactor. After precipitation, the samples were filtered, washed with isopropanol, dried at a temperature of 80 °C for 12 h, and calcined at a temperature of 600 °C for 2 h. The final Eu concentration in the samples was 0.5, 1 and 2 mol.%.

Then, the obtained nanopowders for introducing an additional number of vacancies were subjected to reductive annealing in a hydrogen stream at a temperature of 200 °C for 2 h (designated as SnO_2_:*x*Eu + H_2_, where *x* = 0.5, 1 or 2).

The X-ray diffraction patterns of the samples were obtained on an XPertProMPD diffractometer (PANanalytical, Malvern, UK) in CuKα 1.5405 Å radiation. The crystallite size (coherent scattering region, CSR) was determined by the Scherrer method.

XPS was used to measure survey spectra in the binding energies range of 0–1400 eV and high-energy resolved core levels and valence band (VB) spectra with the help of a PHI XPS 5000 VersaProbe spectrometer (ULVAC-Physical Electronics, Chanhassen, MN, USA) with a spherical quartz monochromator and an energy analyzer working in the range of binding energies from 0 to 1500 eV. The energy resolution was ΔE ≤ 0.5 eV. The samples were kept in the vacuum chamber for 60 min prior to the experiments and were measured at a pressure of 10^−7^ Pa. The X-ray power on the sample did not exceed 50 W, and the beam diameter was 200 μm. The obtained spectra were finally processed using the ULVAC-PHI MultiPak 9.9.0.8 software. The residual background was taken into account using the Shirley method. The energy position was calibrated by the carbon core level C 1*s* (*E* = 285 eV).

The optical diffuse reflectance spectra were measured at room temperature using a Shimadzu UV-2450 spectrophotometer (Shimadzu, Kyoto, Japan) with an ISR-2200 (220–850 nm) integrating sphere. BaSO_4_ was used as a reference. The optical absorption spectra were obtained by the conversion of the diffuse reflectance spectra to a function *F*(*R*) proportional to the absorption coefficient by the Kubelka–Munk equation: *F*(*R*) = (1 − *R*)^2^/2*R*, where *R* is the diffuse reflectance coefficient. The spectra of pulsed cathodoluminescence (PCL) were measured on a KLAVI-R setup equipped with a RADAN pulsed electron gun (pulse duration 2 ns, electron energy 150 keV, the current density in a pulse 150 A/cm^2^) and a luminescence recorder based on a CCD sensor with an electron–optical converter (the measurement range is 350–800 nm).

## 3. Results and Discussions

**Figure 1:**
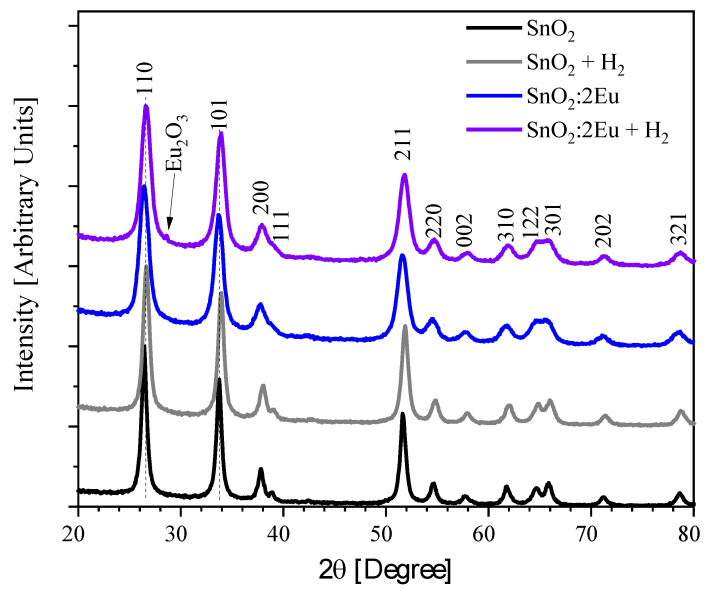
XRD patterns of SnO_2_:*x*Eu and SnO_2_:*x*Eu + H_2_ samples.

According to the X-ray phase analysis presented in Figure 1, the synthesized samples are identified as SnO_2_ with rutile-type crystal structure. Also, adding Eu leads to distortion of the host lattice, which is evidenced by peaks broadening. Note that the (110) and (101) peaks are slightly shifted to lower diffraction angles for the doped sample. But followed annealing returns them to the higher-angle positions. The parameters of lattices are summarized in Table 1. It could be seen that doping and annealing reflected in increased cell parameters. The possible reason for this is differences in ionic radii of Sn^4+^ (0.076 nm) and Eu^3+^ (0.095 nm) [24]. The estimated sizes of CSR indicate the decreasing in size of nanoparticles after doping (from 16 to 9 nm). Such a decrease in particle size will also be accompanied by distortion of the initial host-lattice. Note that a decrease in particle size along with an increase in cell volume will be accompanied by an increment in surface area. Decreasing trends of average crystallite size of SnO_2_ nanoparticles have been reported earlier for Co-doped systems with increasing dopant content [23]. The authors assumed that the decrease can be explained from the perspective of growth kinetics involved during the nucleation process of the nanocrystals [25].

Also, an increase in the cell volume has been previously explained either on the basis of a difference in ionic radii, or a difference in ionic charge [26,27], or a distortion of the coordination geometry of the host cation on doping [28]. Because in our case it could be seen a joint increase in lattice parameters, we can conclude changes in cell volume are caused by differences in ionic radii. On the other hand, different coordination of host cation and dopant will reflect the presence of a larger free space, which may be due to the formation of vacancies in the oxygen sublattice.

It is worth noting that a new diffraction peak appeared after annealing in hydrogen in the sample with 2 mol.% Eu. This peak is attributed to the formation of the Eu_2_O_3_ phase and indicates that for successful forming of DMS where properties are regulated by uniformly distributed impurity atoms but not the secondary phase, we should not increase concentration above this limit.

**Figure 2:**
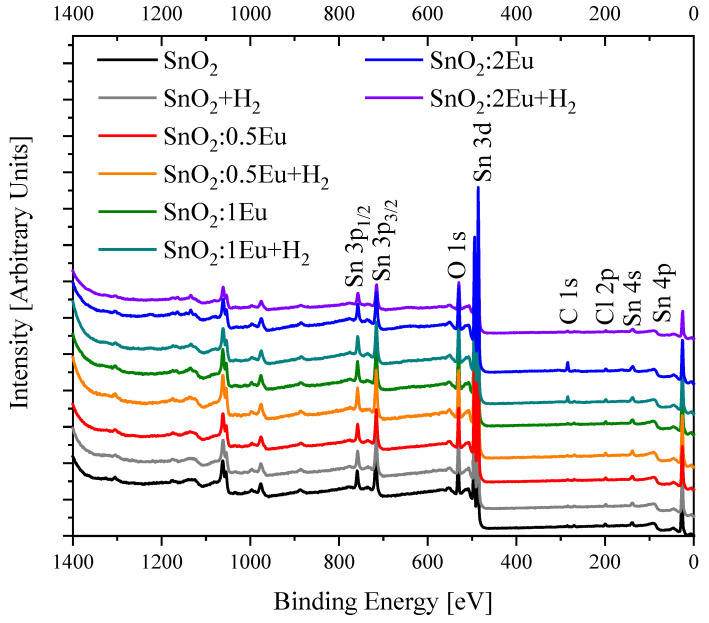
XPS Survey spectra of SnO_2_:*x*Eu and SnO_2_:*x*Eu + H_2_ samples.

XPS Survey spectra presented in Figure 2 show signals only from Sn, O (lattice), Eu (dopant), and C (adventitious carbon). Also, we note a weak Cl 2p peak that arises from the SnCl_4_ precursor. Surface atomic concentrations calculated from the XPS Survey are presented in Table 2. It could be seen from Figure 2 and Table 2 that doping and annealing lead to a small decrease in the O/Sn ratio. It could be induced by the rising number of oxygen vacancies due to substitution of Sn^4+^ by Eu^3+^ or Eu^2+^ and annealing in oxygen-deficient atmosphere.

**Figure 3:**
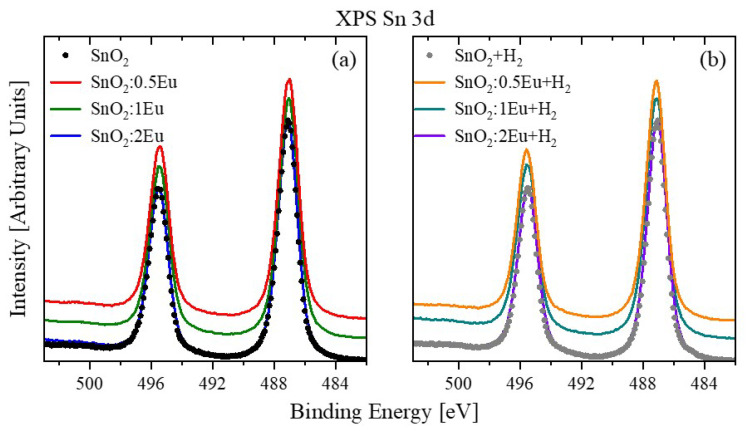
XPS Sn 3*d* spectra of SnO_2_:*x*Eu (**a**) and SnO_2_:*x*Eu + H_2_ (**b**) samples.

The Sn 3*d* spectra for all samples (Figure 3) show one doublet with an energy splitting of 8.40 eV for as-prepared samples and 8.43 eV for annealed ones. Peak Sn 3*d*_5/2_ has a symmetrical shape and the maximum at 487.04 eV and 487.12 eV for the as-prepared and annealed samples, respectively. In this case, there is no significant effect of doping or annealing in hydrogen on the tin sublattice. Some shift of the Sn 3*d*_5/2_ peak to the high-energy region can be caused only by small distortions of the local environment of Sn^4+^ ions caused by non-stoichiometry due to a decrease in the O/Sn ratio (see Table 2) as a result of annealing in an oxygen-deficient atmosphere. There is no decrease in the effective charge of tin, which indirectly indicates the absence of Sn^2+^. It is known that directly in the spectra of Sn 3*d*_5/2_ it is very difficult to distinguish between Sn^2+^ and Sn^4+^ because their positions are close [29]. For this reason it is necessary to measure the spectra of the VB, which will be discussed below.

**Figure 4:**
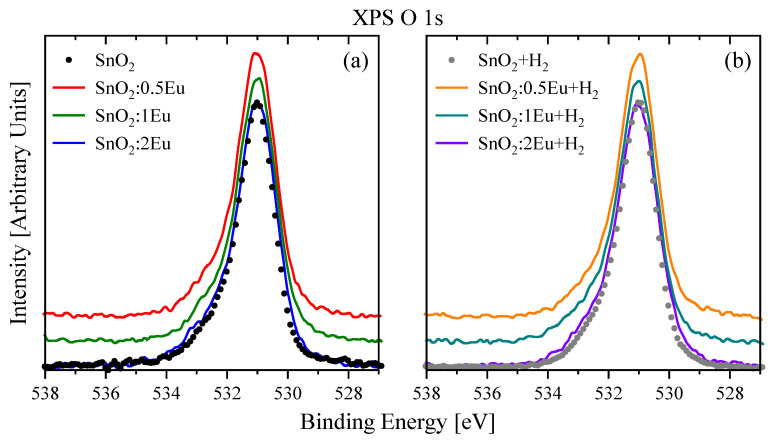
XPS O 1*s* spectra of SnO_2_:*x*Eu (**a**) and SnO_2_:*x*Eu + H_2_ (**b**) samples.

**Figure 5:**
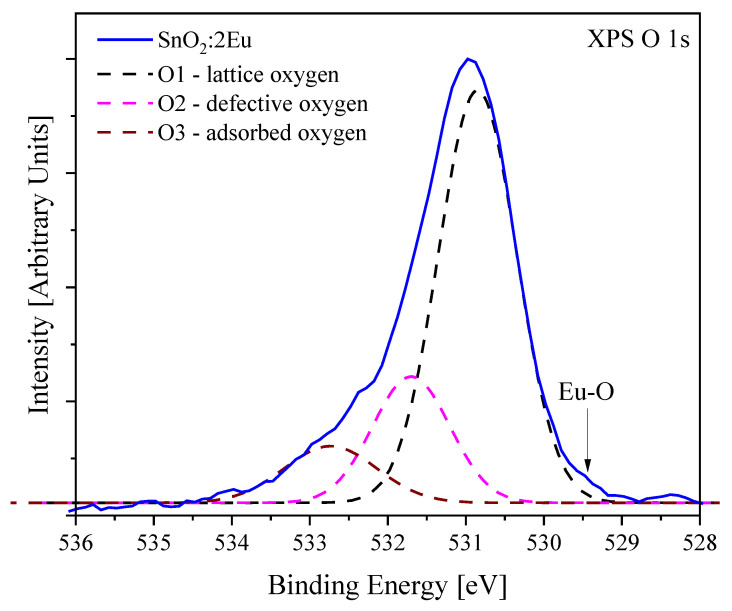
Example of XPS O 1*s* spectra fitting by 3 components: O1—lattice oxygen (530.8 eV), O2—defective oxygen (531.7 eV) and O3—adsorbed oxygen (532.8 eV).

Taking into account the arguments discussed above regarding the defects and distortions of the oxygen lattice, it is important to consider the high-resolution O 1*s* spectra (Figure 4). The spectra of all samples show a non-elementary shape with broadening in the high binding energies region. The main peak is located in the region of 531.02 eV and is in good agreement with the energy position for the O–Sn bond in SnO_2_ [30,31]. At the same time, the appearance of “defective” non-lattice oxygen is known for several oxides [31,32,33]. The signal from these oxygen atoms is located between the peaks of lattice and adsorbed oxygen. Moreover, in Ref. [33], the appearance of a defective peak in SnO_2_ is associated with the formation of [SnO_6_] octahedra. At the same time, in our work, unlike Ref. [33], we do not see noticeable shifts in the Sn 3*d* spectra. Therefore, there is no reason to talk about the formation of [SnO_6_] octahedra. Nevertheless, adhering to the concept of “defective” oxygen, we also deconvoluted O 1*s* spectra into three peaks: lattice oxygen—O1 (530.8 eV), “defective” oxygen—O2 (531.7 eV) and adsorbed oxygen—O3 (532.8 eV). An example of such a fitting is shown in Figure 5, and the peak area ratio is summarized in Table 3. On the one hand, annealing in hydrogen leads only to a slight increase in defective oxygen, which may be associated with the formation of oxygen non-stoichiometry and manifests itself in the Sn 3*d* spectra. On the other hand, the introduction of europium leads to a noticeable decrease in the content of defective oxygen, which is apparently due to the formation of Eu–O bonds (529 eV [34]), which cause a redistribution of the oxygen environment near tin due to the difference in the valence of tin (4+) from europium (2+ or 3+). Thus, europium can locate in the area near “defective” oxygen and lead to a change in O2 peak intensity due to an increasing of the O1 component, to which the Eu–O bond will contribute. In addition, in Ref. [31], it was suggested that oxygen is caused by surface O^2−^ ions from the SnO_2_ lattice, oxygen vacancy, interstitial oxygen, or oxygen antisite in the oxygen-deficient regions, which is consistent with the possible influence of europium on such areas. We emphasize that the annealing of the SnO_2_:2Eu sample leads to a decrease in the O2 peak, which is apparently due to the appearance of the secondary Eu_2_O_3_ phase with the formation of a larger number of Eu–O bonds.

**Figure 6:**
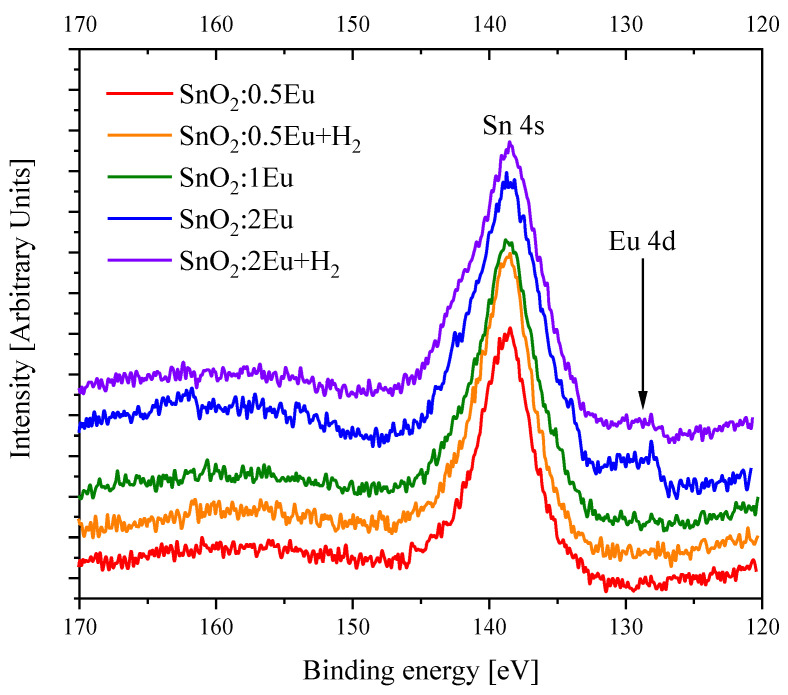
XPS Eu 4*d* spectra (overlapped with Sn 4*s*) of SnO_2_:*x*Eu and SnO_2_:*x*Eu + H_2_ samples.

**Figure 7:**
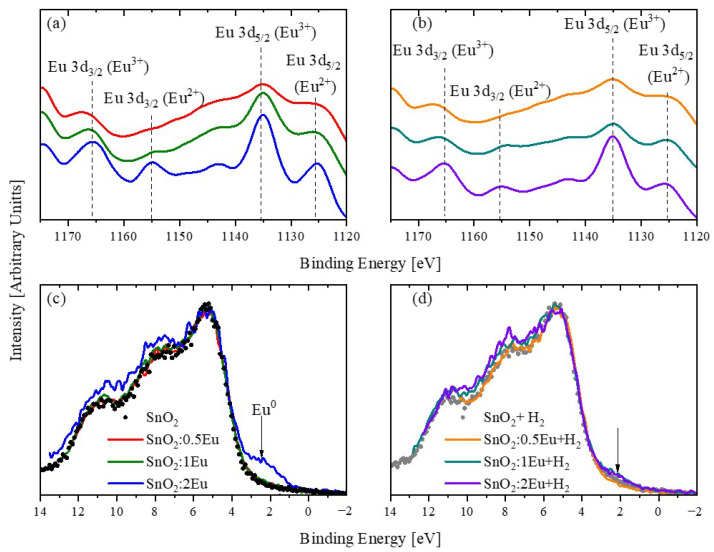
XPS Eu 3*d* (**a**,**b**) and valence band (**c**,**d**) spectra of SnO_2_:*x*Eu (**a**,**c**) and SnO_2_:*x*Eu + H_2_ (**b**,**d**) samples.

The XPS spectra of Eu 4*d* and Eu 3*d* are shown in Figure 6 and Figure 7, respectively. Note that the Eu 4*d* peak strongly overlapped with the signal from the Sn 4*s* level, which does not allow one to draw conclusions about the presence of Eu^3+^. However, even in such a situation, it is seen that the introduction of 2 mol.% europium leads to the appearance of a low-energy feature in the region of 128 eV, which corresponds to the appearance of Eu^2+^ [34,35]. Note that annealing leads to a decrease in the relative intensity of the Eu^2+^ signal in the XPS Eu 4*d* spectra.

To clarify the possibility of the appearance of Eu^3+^, the XPS Eu 3*d* spectra were measured (Figure 7a,b). The spectra of all samples demonstrate the appearance of signals in the region of 1135 eV and 1166 eV, which corresponds to the appearance of trivalent europium [31,34,35,36]. An increase in the europium concentration leads to the appearance of peaks in the region of 1126 eV and 1155 eV, which confirms the appearance of Eu^2+^ [34,35] and is consistent with the data in Figure 6. Moreover, with an increase in the concentration of europium, an increase in the intensity of the peak of Eu^2+^ is also observed. On the other hand, annealing leads to a general decrease in the intensity of both the Eu^2+^ and Eu^3+^ lines, although for the latter this effect is not so pronounced. We assume that annealing leads to a redistribution of the oxygen environment of Eu, which leads, among other things, to distortions in the oxygen subsystem of the tin dioxide lattice and manifests itself in a change in the intensity of the O2 peak in Figure 4 and Figure 5. In addition, a decrease in the Eu^2+^ intensity and a change in the defects in the oxygen sublattice of tin dioxide are in good agreement with the appearance of the secondary Eu_2_O_3_ phase evidenced by XRD in the annealed SnO_2_:2Eu + H_2_ sample (Figure 1). At the same time, an increase in imperfection with the introduction of europium and annealing is also evidenced by a change in the lattice parameters (Table 1).

XPS spectra of the VB are presented in Figure 7c,d. By analogy with Yb-doped SnO_2_, one could expect a low-energy shift in the VB edge and a redistribution of band intensities in the regions of 5 eV (O 2*p*-derived levels), 8 eV (hybridization between Sn 5*p* and O 2*p* orbitals), and 11 eV (Sn 5*s*—O 2*p* bonding states) [15]. However, the introduction of even 2 mol.% of europium leads only to a slight increase in the intensity of the last two features and does not reflect in a shift in the VB edge. The most noticeable change is the appearance of a signal in the region of 2 eV, which may indicate the appearance of metallic europium. Note that the position of the 3*d* core levels of Eu^0^ and Eu^2+^ (1126 eV) [35,37] is almost identical and does not allow us to separate them in the spectra of core levels. In addition, it is known [29] that the VB spectra of SnO and SnO_2_ differ significantly in contrast to Sn 3*d*. Moreover, a distinctive feature of SnO is the presence of a maximum in the region of 2 eV. Thus, the introduction of 2 mol.% of europium can result in the appearance of metallic europium, as well as in the appearance of highly defective regions close in stoichiometry to SnO. The first scenario agrees well with the well-known feature of europium to crystallize well and form secondary phases when a certain concentration limit is reached. The second is confirmed by significant defectiveness samples. Despite the fact that XRD does not show the presence of metallic europium, we assume that the appearance of this feature is associated precisely with the formation of Eu–Eu bonds and that the band at 1126 eV in the XPS spectra of Eu 3*d* is due to Eu^0^ rather than Eu^2+^. This assumption was made on the basis that annealing leads to the disappearance of this feature, while the spectra of core levels of Sn 3*d* and O 1*s* indicate an increase in imperfection. In addition, the disappearance of this feature as a result of annealing can be associated with the appearance of a secondary Eu_2_O_3_ phase, which is accompanied by the formation of strongly non-stoichiometric SnO_2−*x*_ areas.

**Figure 8:**
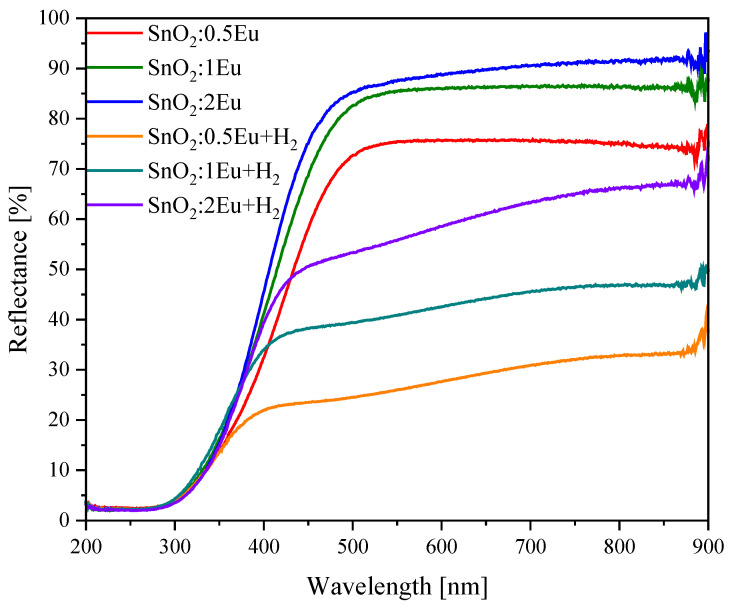
Diffuse reflectance spectra of the SnO_2_:*x*Eu and SnO_2_:*x*Eu + H_2_ samples.

**Figure 9:**
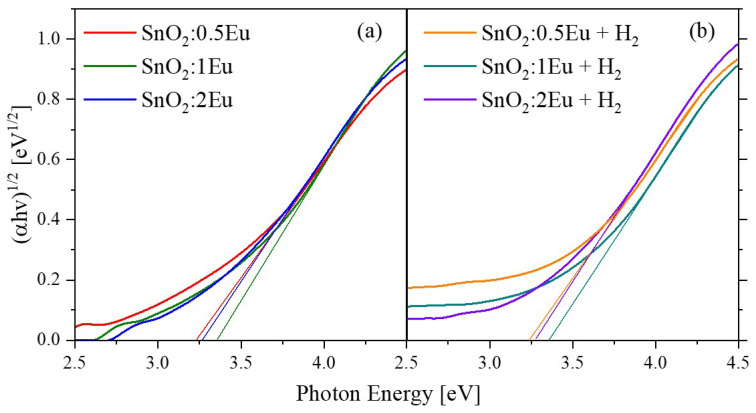
Optical absorption edge in Tauc plot of SnO_2_:*x*Eu (**a**) and SnO_2_:*x*Eu + H_2_ (**b**) samples.

Changes in VB, in particular the appearance of strongly defective regions close to the stoichiometry of SnO, should inevitably manifest themselves in optical properties. For this purpose, diffuse reflection spectra were studied (Figure 8). It is seen that an increase in the content of europium leads to a significant increase in reflection in the visible region. One of the reasons for this behavior may be the appearance of europium in the metallic state, which is in good agreement with changes in the Eu 3*d* and XPS VB spectra. Annealing leads to a decrease in the Eu^0^ content and manifests itself in a decrease in reflection. At the same time, a significant decrease in reflection is observed as a result of annealing of the samples in a reducing atmosphere. The optical absorption coefficient was calculated using the Kubelka–Munk model. The bandgap energies of semiconductors close to the band edge can be expressed by the Tauc plot [38]. It can be seen from Figure 9 that Eu doping and annealing do not dramatically change the absorption edge. The calculated band gap values are summarized in Table 4. It is known that oxygen-deficient SnO_2_ leads to reducing the band gap [39]. Also, a decreasing particle size leads to an increasing band gap [40]. We attribute the small changes seen after doping up to 1 mol.% of Eu to a decrease of particle sizes. And the following decreasing of band gap could be induced by higher lattice disorder and/or participation of secondary Eu_2_O_3_ phase.

**Figure 10:**
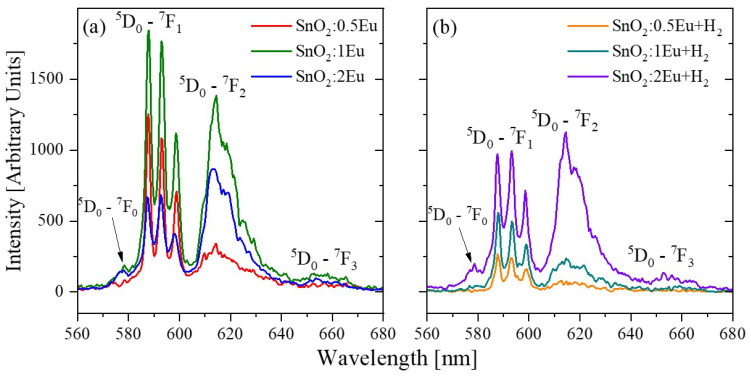
PCL spectra of SnO_2_:*x*Eu (**a**) and SnO_2_:*x*Eu + H_2_ (**b**) samples.

To further study the Eu local environment, the pulse cathodoluminescence (PCL) was measured (Figure 10). Luminescence spectra of Eu^3+^ centers in crystalline SnO_2_ matrices may provide important information about its substitutional position with different crystalline fields in the host [17,40]. The ^5^D_0_→^7^F_1_ are magnetic dipole allowed transitions and therefore are not affected by nearby structural changes. However, the ^5^D_0_→^7^F_2_ is electric dipole-ruled and hypersensitive to the local crystalline field [40]. The ratio between these two transitions (^5^D_0_→^7^F_2_/^5^D_0_→^7^F_1_) is called the asymmetric ratio (*R*_21_, Table 4) and can be used for conclusions about the location distribution of the Eu^3+^ in the SnO_2_ matrix [41]. The lower value of *R*_21_ shows that the Eu^3+^ may be located at no distorted site substitute for Sn^4+^ with oxygen vacancy formation to guarantee the charge neutrality of the solid. It is a case of SnO_2_:0.5Eu samples where the number of dopant atoms is relatively low and lattices are not disordered. Differently, the increase in the asymmetric ratio (SnO_2_:2Eu samples, Table 4) indicates that more Eu^3+^ species are located mainly at distorted sites at the SnO_2_ matrix. This conclusion is also supported by the distortion of the unit cell, which is indicated by the changing of lattice parameters (see Table 1). On the other hand, the PCL of SnO_2_:2Eu is quenched due to the higher dopant concentration. It should be noted that annealing tends to quench luminescence, which could be caused by non-radiative relaxation through a higher number of oxygen vacancies. Moreover, the annealing in hydrogen reduces the asymmetric ratio *R*_21_ (Table 4) that is also confirmed by the Eu^3+^ local environment changes by the oxygen vacancy creation. In addition, the study of PCL spectra confirms the possibility of the formation of highly non-stoichiometric regions, which can cause an increase in intensity at the edge of the valence band (Figure 7), which explains the incomplete disappearance of this feature after annealing.

Thus, an increase in the europium content and annealing lead to a larger lattice distortion due to the creation of a larger number of oxygen vacancies, disordered regions, and a decreasing of crystallite sizes.

## 4. Conclusions

In conclusion, we successfully synthesized nanocrystalline SnO_2_ particles doped with 0.5, 1, and 2 mol.% Eu. Studies show that the introduction of europium leads to the formation of a significant number of vacancies due to the substitution of Sn^4+^→Eu^3+^. Furthermore, an increase in the concentration of europium leads to the appearance of Eu^2+^ and Eu^0^, which causes lattice distortion and the formation of strongly defective regions. The used annealing in a reducing atmosphere, on the one hand, caused the appearance of additional oxygen vacancies. On the other hand, it led to the transfer of a part of Eu^2+^ ions to Eu^3+^ and the destruction of Eu–Eu bonds with the formation of a secondary Eu_2_O_3_ phase. Luminescent measurements confirm the formation of highly defective regions in samples with a high europium content.

## Figures and Tables

**Table 1:** Lattice parameters *a* and *c*, the size of the CSR and cell volume.

Sample	*a* (Å)	*c* (Å)	*c*/*a*	CSR (nm)	Volume (Å^3^)
SnO_2_	4.73440	3.18380	0.67248	16	71.36354
SnO_2_ + H_2_	4.73773	3.18662	0.67261	15	71.52704
SnO_2_:2Eu	4.74302	3.18959	0.67248	9	71.75381
SnO_2_:2Eu + H_2_	4.74634	3.19217	0.67255	9	71.9123

**Table 2:** Surface composition of SnO_2_:*x*Eu and SnO_2_:*x*Eu + H_2_ samples (in at. %).

Sample	Sn	O	C	Eu	Cl	O/Sn
SnO_2_	32.9	61	3.9		2.2	1.85
SnO_2_ + H_2_	33.4	60.5	4.1		2	1.81
SnO_2_:0.5Eu	33.5	61.5	2.5	0.3	2.1	1.83
SnO_2_:0.5Eu + H_2_	33.6	60.4	2.4	0.3	3.3	1.79
SnO_2_:1Eu	33.5	60.7	2.6	0.7	2.5	1.81
SnO_2_:1Eu + H_2_	28.1	51	18.1	0.7	2.1	1.81
SnO_2_:2Eu	25.3	47.3	24.5	1.1	1.8	1.87
SnO_2_:2Eu + H_2_	32.4	59.4	4.3	1.6	2.3	1.83

**Table 3:** Relative content (in %) of O 1*s* components of SnO_2_:*x*Eu and SnO_2_:*x*Eu + H_2_ samples: O1—lattice oxygen, O2—defective oxygen and O3—adsorbed oxygen.

Sample	O1	O2	O3
SnO_2_	80.6	17	2.4
SnO_2_ + H_2_	80.6	17.2	2.2
SnO_2_:0.5Eu	87.3	9.2	3.5
SnO_2_:0.5Eu + H_2_	81.6	14.4	4
SnO_2_:1Eu	89	9.3	1.7
SnO_2_:1Eu + H_2_	85.1	13.4	1.5
SnO_2_:2Eu	83.3	13.6	3.1
SnO_2_:2Eu + H_2_	85.5	12.2	2.3

**Table 4:** Energy gap (*E_g_*) and asymmetric ratio (*R*_21_) SnO_2_:*x*Eu and SnO_2_:*x*Eu + H_2_ samples.

Sample	SnO_2_:0.5Eu	SnO_2_:1Eu	SnO_2_:2Eu	SnO_2_:0.5Eu + H_2_	SnO_2_:1Eu + H_2_	SnO_2_:2Eu + H_2_
***E_g_* (eV)**	3.23	3.36	3.27	3.23	3.36	3.28
***R*_21_**	0.71	1.25	1.97	0.60	0.88	1.61

## Data Availability

Data are contained within the article.
